# Supplementary material for: Evaluation of outcome reporting in clinical trials of physiotherapy in bronchiectasis: The first stage of core outcome set development
Source: PLoS One. 2023 Mar 16;18(3):e0282393. doi: 10.1371/journal.pone.0282393 (PMC10019700; doi:10.1371/journal.pone.0282393)
Supplement: S1 Appendix — (DOCX) [file pone.0282393.s001.docx]

**Appendix 1**

**Search strategy** *Ovid Medline*

**Search strategy for physiotherapy**

1 exp Physical Therapy Modalities/
2 (physiotherap* or physical therap* or physical treatment*).tw.
3 exp Respiratory Therapy/
4 exp Positive Pressure Respiration/
5 Breathing Exercises/
6 Vibration/
7 (patient* adj3 (postur* or position*)).tw.
8 (body adj3 (postur* or position* or lateral)).tw.
9 (oscillat* or vibrat* or percuss* or huff*).tw.
10 ((chest or thora*) adj3 (clap* or shak* or compress*)).tw.
11 (cough* adj2 (directed or maneuver* or manoeuver* or techniqu*)).tw.
12 positive pressure ventilation*.tw.
13 positive expiratory pressure*.tw.
14 electrostimulat*.tw.
15 massag*.tw.
16 ((respirat* or ventilat*) adj2 muscle train*).tw.
17 ((postur* or autogenic) adj2 drain*).tw.
18 (breath* adj2 (control* or techni* or train* or exercis* or "active cycle")).tw.
19 ((forced or slow or prolonged or increas* or control*) adj2 (exhal* or expir*)).tw.
20 flutter.tw.
21 (incentive adj2 (inspiromet* or spiromet*)).tw.
22 eltgol.tw.
23 or/1‐22

**Search strategy for bronchiectasis**

24 exp Bronchiectasis/
25 bronchiect$.ti,ab.
26 or/24 - 25

27 23 and 26

**Search strategy for randomized controlled trials**

28 (controlled clinical trial or randomized controlled trial).pt.
29 (randomized or randomised).ab,ti.
30 placebo.ab,ti.
31 randomly.ab,ti.
32 trial.ab,ti.
33 groups.ab,ti.
34 or/27‐32

**Combination of above intervention, condition and study design, with search limiters**
35 Animals/
36 Children/

37 35 not (35 - 36)
38 34 not 37

39 27 and 38
