# Supplementary material for: Evaluation of outcome reporting in clinical trials of physiotherapy in bronchiectasis: The first stage of core outcome set development
Source: PLoS One. 2023 Mar 16;18(3):e0282393. doi: 10.1371/journal.pone.0282393 (PMC10019700; doi:10.1371/journal.pone.0282393)
Supplement: S2 Appendix — (DOCX) [file pone.0282393.s002.docx]

# Appendix 2

The full list of outcomes

| Core area | Domain | Outcome |
| --- | --- | --- |
| Clinical/Physiological outcomes | Blood and lymphatic system outcomes | 1. Blood cell count |
|  | Cardiac outcomes | 1. Heart rate |
|  | General outcomes | 1. Body weight |
|  |  | 1. Body composition |
|  |  | 1. Disease severity |
|  | Infection and infestation outcomes | 1. Sputum neutrophil elastase level |
|  |  | 1. Nasal lavage fluid |
|  |  | 1. Exhaled breath condensate |
|  |  | 1. Cytokines in Exhaled breath condensate |
|  |  | 1. pH level in Exhaled breath condensate |
|  |  | 1. Exhaled Nitric Oxide Fraction (FENO) |
|  |  | 1. Cytokines in nasal lavage fluid |
|  |  | 1. Tumor Necrosis Factor-α (TNF-α) in nasal lavage fluid |
|  |  | 1. Interleukins IL-6 and IL-10 in nasal lavage fluid |
|  |  | 1. Blood inflammation markers |
|  |  | 1. Sputum inflammation markers |
|  | Musculoskeletal and connective tissue outcomes | 1. Muscle strength |
|  | Respiratory, thoracic and mediastinal outcomes | 1. Oxygen saturation (SPO2) |
|  |  | 1. Airway resistance |
|  |  | 1. Respiratory rate |
|  |  | 1. Respiratory resistance |
|  |  | 1. Lung sounds |
|  |  | 1. Non-invasive ventilation resistance rate |
|  |  | 1. Arterial blood gases |
|  |  | 1. Alveolar – arterial oxygen gradient |
|  | Respiratory, thoracic and mediastinal outcomes  a. Respiratory Muscle function | 1. Respiratory muscle strength |
|  |  | 1. Respiratory muscle endurance |
|  |  | 1. Maximal Expiratory Pressure (PEmax) |
|  |  | 1. Maximal Inspiratory Pressure (PImax) |
|  | Respiratory, thoracic and mediastinal outcomes  b. Lung function | 1. Pulmonary function (spirometry) |
|  |  | 1. Forced expiratory volume in one second (FEV1) |
|  |  | 1. Forced vital capacity (FVC) |
|  |  | 1. Maximal mid-expiratory flow (MMEF) |
|  |  | 1. FEV1 /FVC |
|  |  | 1. forced expiratory flow between 25 and 75% of the FVC (FEF25-75%) |
|  |  | 1. peak expiratory flow rate (PEFR) |
|  |  | 1. Inspiratory capacity (IC) |
|  |  | 1. Vital capacity (VC) |
|  |  | 1. Total Lung Capacity (TLC) |
|  |  | 1. Lung volumes |
|  |  | 1. Residual Volume (RV) |
|  |  | 1. Lung carbon monoxide transfer factor (TLCO) |
|  |  | 1. Percentages of predicted spirometry values |
|  |  | 1. Ventilation inhomogeneity/Lung Clearance Index (LCI) |
|  | Respiratory, thoracic and mediastinal outcomes  c. sputum | 1. Sputum weight |
|  |  | 1. Sputum volume |
|  |  | 1. Sputum dry weight |
|  |  | 1. Sputum production |
|  |  | 1. In vitro mucociliary transport |
|  |  | 1. Sputum microbiology |
|  |  | 1. Secretion purulence |
|  |  | 1. Sputum Viscosity |
|  |  | 1. Sputum Elasticity |
|  |  | 1. In vitro sputum cough clearability |
|  |  | 1. The contact angle of sputum |
|  |  | 1. Secretion adhesiveness |
|  |  | 1. Sputum cell count |
|  |  | 1. In vivo mucociliary transport |
|  |  | 1. Secretion surface properties and appearance |
|  |  | 1. Sputum colour |
|  | Respiratory, thoracic and mediastinal outcomes d. patient reported symptoms | 1. Breathlessness |
|  |  | 1. Number of coughs |
|  |  | 1. Cough symptoms |
|  |  | 1. Fatigue |
|  |  | 1. Change in respiratory symptoms |
|  |  | 1. Sputum symptoms |
|  | Respiratory, thoracic and mediastinal outcomes e. exacerbations | 1. Time to first exacerbation |
|  |  | 1. Exacerbation frequency |
| Life impact | Physical functioning | 1. Physical activity and fitness level |
|  |  | 1. Functional Exercise capacity |
|  |  | 1. Six minute walk distance |
|  |  | 1. Maximum exercise tolerance |
|  |  | 1. Endurance walk capacity |
|  |  | 1. Maximal treadmill exercise capacity |
|  |  | 1. Energy cost in walking |
|  |  | 1. Sleep quality |
|  | Emotional functioning/wellbeing | 1. Anxiety and depression |
|  |  | 1. Confidence and self-efficacy |
|  | Cognitive functioning | 1. Cognitive loss |
|  | Global quality of life | 1. health-related quality of life (HRQOL) |
|  |  | 1. Cough-related quality of life |
|  |  | 1. Quality adjusted life years (QALYs) |
|  | Perceived health status | 1. General health status |
|  | Delivery of care  a. patient reported experience | 1. Patient preference |
|  |  | 1. Participant satisfaction |
|  |  | 1. Adherence to treatment |
|  |  | 1. Acceptability and Tolerance of treatment |
|  |  | 1. Comfort of technique |
|  |  | 1. Perceived benefits obtained |
|  |  | 1. General perceptions regarding interventions |
|  |  | 1. Patient perceived effectiveness |
|  |  | 1. Illness perception |
|  |  | 1. Self-rated ability to manage bronchiectasis |
|  | Delivery of care  b. intervention monitored parameters | 1. Number of sets performed during session |
|  |  | 1. Symptoms developed during intervention |
| Resource use | Hospital admission | 1. Number of urgent hospital admissions |
|  |  | 1. Number of inpatient hospital days |
|  |  | 1. Number of Intensive Care Unit (ICU) admissions |
|  |  | 1. Number of ICU days |
|  | Use of healthcare resources | 1. Number of urgent/unplanned outpatient visits |
|  |  | 1. Self-rated healthcare use |
|  | Need for further intervention | 1. Need for invasive mechanical ventilation. |
|  |  | 1. Antibiotics use |
| Adverse events/effects | Adverse events/effects | 1. Side effects |
