# Supplementary material for: Evaluation of outcome reporting in clinical trials of physiotherapy in bronchiectasis: The first stage of core outcome set development
Source: PLoS One. 2023 Mar 16;18(3):e0282393. doi: 10.1371/journal.pone.0282393 (PMC10019700; doi:10.1371/journal.pone.0282393)
Supplement: S3 Appendix — (DOCX) [file pone.0282393.s003.docx]

# Appendix 3

Frequency of reporting per outcome, calculated by number of trials and protocol that reported outcome

| Outcome | Frequency |
| --- | --- |
| 1. Blood cell count | 1 |
| 1. Heart rate | 3 |
| 1. Body weight | 1 |
| 1. Body composition | 1 |
| 1. Disease severity | 3 |
| 1. Sputum neutrophil elastase level | 1 |
| 1. Nasal lavage fluid | 1 |
| 1. Exhaled breath condensate | 2 |
| 1. Cytokines in Exhaled breath condensate | 1 |
| 1. pH level in Exhaled breath condensate | 1 |
| 1. Exhaled Nitric Oxide Fraction (FeNO) | 2 |
| 1. Cytokine level in nasal lavage fluid | 2 |
| 1. Tumor Necrosis Factor-α (TNF-α) in nasal lavage fluid | 2 |
| 1. Interleukin 6 (IL-6) and Interleukin 10 (IL-10) in nasal lavage fluid | 2 |
| 1. Blood inflammation markers | 4 |
| 1. Sputum markers of inflammation | 1 |
| 1. Muscle strength | 5 |
| 1. Oxygen saturation (SPO2) | 10 |
| 1. Airways resistance | 4 |
| 1. Respiratory rate | 1 |
| 1. Respiratory resistance | 1 |
| 1. Lung sounds | 1 |
| 1. Non-invasive ventilation resistance rate | 1 |
| 1. Arterial Blood gas | 2 |
| 1. Alveolar – arterial oxygen gradient | 1 |
| 1. Respiratory muscle strength | 3 |
| 1. Respiratory muscle endurance | 1 |
| 1. Maximal expiratory pressure (PEmax) | 8 |
| 1. Maximal inspiratory pressure (PImax) | 8 |
| 1. Pulmonary function | 27 |
| 1. Forced expiratory volume in one second (FEV1) | 17 |
| 1. Forced vital capacity (FVC) | 14 |
| 1. Maximal mid-expiratory flow (MMEF) | 2 |
| 1. FEV1 /FVC | 3 |
| 1. Forced expiratory flow between 25 and 75% of the FVC (FEF25-75%) | 4 |
| 1. Peak expiratory flow rate (PEFR) | 3 |
| 1. Inspiratory capacity (IC) | 1 |
| 1. Vital capacity (VC) | 1 |
| 1. Total lung capacity (TLC) | 1 |
| 1. Lung volumes | 4 |
| 1. Residual volume (RV) | 1 |
| 1. Lung carbon monoxide transfer factor (TLCO) | 1 |
| 1. Percentages of predicted spirometry values | 1 |
| 1. Ventilation inhomogeneity, Lung clearance index (LCI) | 1 |
| 1. Sputum weight | 15 |
| 1. Sputum volume | 12 |
| 1. Secretions dry weight | 6 |
| 1. Sputum production | 3 |
| 1. In vitro mucociliary transport | 4 |
| 1. Sputum microbiology | 3 |
| 1. Secretion purulence | 3 |
| 1. Sputum Viscosity | 3 |
| 1. Sputum Elasticity | 3 |
| 1. In vitro sputum cough clearability | 3 |
| 1. The contact angle of sputum | 3 |
| 1. Secretion adhesiveness | 2 |
| 1. Sputum cell count | 2 |
| 1. In vivo Mucociliary transport | 2 |
| 1. Secretion surface properties and appearance | 2 |
| 1. Sputum colour | 1 |
| 1. Breathlessness | 18 |
| 1. Number of coughs | 4 |
| 1. Cough symptoms | 5 |
| 1. Fatigue | 3 |
| 1. Change in respiratory symptoms | 3 |
| 1. Sputum symptoms | 2 |
| 1. Time to first exacerbation | 3 |
| 1. Exacerbation frequency | 7 |
| 1. Physical activity level | 4 |
| 1. Functional Exercise capacity | 9 |
| 1. Six minute walk distance | 10 |
| 1. Maximum exercise tolerance | 4 |
| 1. Endurance walk capacity | 4 |
| 1. Maximal treadmill exercise capacity | 2 |
| 1. Energy cost in walking | 2 |
| 1. Sleep quality | 1 |
| 1. Anxiety and depression | 4 |
| 1. Confidence and self-efficacy | 1 |
| 1. Cognitive loss | 1 |
| 1. health-related quality of life (HRQOL) | 26 |
| 1. Cough specific health-related quality of life | 9 |
| 1. Quality adjusted life years (QALYs) | 1 |
| 1. General health status | 4 |
| 1. Patient preference | 1 |
| 1. Participant satisfaction | 1 |
| 1. Adherence to treatment | 12 |
| 1. Acceptability and Tolerance of treatment | 4 |
| 1. Comfort of technique | 3 |
| 1. Perceived benefits obtained | 1 |
| 1. General perceptions regarding interventions | 1 |
| 1. Patient perceived effectiveness | 1 |
| 1. Illness perception | 1 |
| 1. Self-rated ability to manage bronchiectasis | 1 |
| 1. Number of sets performed during session | 1 |
| 1. Symptoms developed during intervention | 1 |
| 1. Number of urgent hospital admissions | 2 |
| 1. Number of inpatient hospital days | 2 |
| 1. Number of Intensive Care Unit (ICU) admissions | 1 |
| 1. Number of ICU days | 1 |
| 1. Number of urgent/unplanned outpatient visits | 3 |
| 1. Self-rated healthcare use | 1 |
| 1. Use of antibiotics | 3 |
| 1. Need for invasive mechanical ventilation. | 1 |
| 1. Adverse events | 2 |
