# Supplementary material for: Evaluation of outcome reporting in clinical trials of physiotherapy in bronchiectasis: The first stage of core outcome set development
Source: PLoS One. 2023 Mar 16;18(3):e0282393. doi: 10.1371/journal.pone.0282393 (PMC10019700; doi:10.1371/journal.pone.0282393)
Supplement: Attachment — Submitted filename: Response to reviewers PONE-D-22-28058.docx [file pone.0282393.s005.docx]

Response to reviewers

Revisions table Manuscript Number: PONE-D-22-28058

| **Reviewer #1** | |
| --- | --- |
| **Comment** | **Response** |
| Reviewer #1: This is an interesting and important study establishing what outcomes are currently reported for clinical trials of physiotherapy for people with bronchiectasis. Some points of clarification are required, and suggestions are detailed below. | We would like to thank you for reviewing the manuscript and valuing the work we have done. Thank you for highlighting errors. Spelling and grammar are now revised throughout the manuscript |
| Abstract Page 2 – define COS in full on line 32. It is not clear what the numbers after lung function, health related quality of life etc mean. Presumably, these are n=, but this perhaps needs to be made clearer to the reader. This also applies within the results section of the manuscript. Was a pre-specified system for classifying into domains used? | All abstract points are now corrected. |
| page 4, line 71 - COS-PHyBE needs to be defined for your readers. | Definition added |
| Page 5, line 88 – needs rephrasing “More specifically, the two main objectives are creating a synthesised long list 89 of outcomes and assessing the variation in outcome reporting.’ | Sentence rephrased:  “More specifically, the two main objectives are (1) creating a synthesised long-list of outcomes reported in literature and (2) assessing the variation in outcome reporting among relevant trials.” |
| Under results and search results, the numbers quoted don’t completely add up – if 1528 remained, then a further 1388 were removed, this leaves 140 full texts rather than 141? | Corrected to 140 |
| Page 9, line 193 – incomplete sentence ‘While 23 193 compared one form of physiotherapy to another by including an active comparator group 194 (table 2).’ | Sentence rephrased: (page 11, line 203)  “Thirteen trials compared physiotherapy to control, sham, or placebo; while 23 trials compared two or more physiotherapy techniques using active comparator groups (table 2).” |
| On page 9, flutter device is described as a technique, but the actual technique is oscillating PEP or oscillatory PEP, the flutter device is just what is used to deliver this technique. This needs to be corrected to accurately reflect the type of technique rather than the device chosen to deliver it. | Sentence rephrased: (page 11, line 206)  “The two most investigated techniques were the active cycle of breathing technique (ACBT) and the Positive Expiratory Pressure devices (PEP).” |
| Not sure what is meant by the three categories of airway clearance techniques? Can the authors elaborate on this? I can see it is later stated in the figure, but it is still not clear what the difference is between airway clearance techniques PEP/OPEP etc and positioning. Wondering if the authors are referring to breathing techniques when they mean airway clearance techniques, following the ERS definition (from 2017). If so, this needs to be made clearer in the text. | Classification of techniques is revised in text and figure 2. |
| In the paragraph of Interventions under results, there is a lack of references when stating 13 trials compared physiotherapy to sham, etc. It would be useful to have links to the relevant studies in this paragraph. Some alterations to grammar are required within the manuscript to improve clarity and readability. | Table 2 is referenced here, and it explains all relevant information |
| On page 10, it is not clear how the decision to choose a unique name presumably based on the COMET taxomony for outcomes with the same meaning was undertaken. Was this done by one author or a consensus? | Explained in methods section (Creating unique outcome long list) page 8 |
| In Table 2, it is not clear how the decision was made for what is the intervention versus the comparison? It implies that the focus of the study was on the intervention, but for some included studies, they compared 3 techniques, with no one technique the main focus. Can the authors provide some further clarity or consider reworking their description of interventions in this table? Also, there is inconsistency within the table, crossover and Crossover both appear – suggest fixing this. | Interventions and comparators are described as reported in the study report.  Crossover spelling is corrected in table 2 |
| In Table 3, some abbreviations are in ( ), others are not – suggest consistency throughout with careful attention to detail. | Corrected |
| Figure 2 – what makes up CPT – as this has previously been PD as well, which would fit under positioning? | Figure 2 is revised to add more clarity |
| Not sure that Figure 3 adds much to the results, it is hard to understand the takeaway message from this? | Figure 3 was removed |
| Correct spelling of Aerobika and consider which devices are registered names and require ® at the end. | Corrected |
| Appendix 2 – consider putting SpO2 in ( ) like the rest of abbreviations. Same for QALYs. Also needs to be adjusted in Appendix 3. | Corrected |
| LCI is mentioned for ventilation homogeneity but needs an explanation in the legend. Same for ICU, IL6, and Il10 and TNF-alpha. This applies in Appendix 2 and 3 | All abbreviations in appendix 2 and appendix 3 are now defined |
| Unfinished sentence – page 11, line 239 ‘Which predicts a continuous problem of research waste in 240 the future [35].’ Same for page 12, line 269 ‘While the European Respiratory Society 270 (ERS) guidelines encouraged researching the effectiveness of physiotherapy in terms of 271 accessibility, patient preference and adherence [10].’ | Corrected |
| Not sure that I would describe crossover designs as inappropriate for physiotherapy studies as sometimes the way they are conducted and the adequate washout period is fine, particularly if the sample size is difficult to achieve for an RCT design. Consider rephrasing here. | Sentenced rephrased:  “Notwithstanding the potential methodological issues of crossover designs for physiotherapy studies,” Page 15 line 260 |
| Page 11 – define CONSORT in full | Definition added page 16 line 276 |
| Authors mention the lack of consensus on important outcomes, they could also refer to research priorities which have been published by the ERS and USA bronchiectasis registry, as a way of drawing on what knowledge or other suggestions exist for outcomes as well, as a broad guide. | We already referred to relevant guidelines from ACCP, ERS and BTS which discussed outcomes in second paragraph page 16. |
| Authors could consider joining 2 paragraphs on page 13, paragraph 2 and 3 together. Same for the paragraphs on page 14, the first 2 could be joined together, as the first paragraph is quite short. | Revised |
| Minor points Page 2, line 37 – add ‘and’ before home exercise program. | Corrected |
| Line 45 – 18 should be written in full at the start of a sentence. | Corrected |
| Page 4 – line 63 – UK should be written in full | Corrected |
| Numbers less than 10 should be written in full? If this is the case for this journal, it needs to be attended to throughout the manuscript. There is inconsistency within the manuscript. | Numbers are revised throughout the manuscript |
| Page 4, ine 84 – replace wasn’t, with was not. | Corrected |
| A mix of tenses are used in the introduction – would be better to describe the aim of the study was ‘was to identify’, given it has already happened. In the methods – commentary on the Full publication, pilot studies, protocols….. are included – should read as were included. Suggest reviewing the overall manuscript to gain consistency in tense use throughout. | Revised throughout the manuscript |
| Page 5 – line 101 – studies should be ‘Studies’ | Corrected |
| Page 6, line 133 – define PRISMA | Definition added |
| Page 7, line 156 ‘two reviewers agreed upon a unique name..’ Consider altering the wording for this sentence to improve clarity. | Corrected |
| Page 8, line 181, unclear why table (2) in written in this way? Same for figure (4)? | Corrected |
| Page 8, line 182 – CCT should be written as (CCT), as it is an abbreviation. | Corrected |
| Typo page 13, line 303 – durnig should read during | Corrected |
| Reviewer #2: |  |
| This is an interesting systematic review, with a strong methodology examining outcome reporting in clinical trials of physiotherapy in bronchiectasis. Some suggestions below outline points requiring further clarification. | We would like to thank you for reviewing the manuscript and valuing the work we have done. Thank you for highlighting errors. Spelling and grammar are now revised throughout the manuscript |
| Abstract, page 2, line 45 – 18 should be written as eighteen at the start of a sentence. Same for line 177 – 74 should be written as Seventy-four… | Both numbers corrected |
| There is a trend in this manuscript to introduce abbreviations prior to introducing their full name. Suggest reversing this order throughout the manuscript. This is evident in the introduction, with reference to COS-PHyBE, but no explanation of what this is. Same for RCTS later in the methods. Other times this is reversed. Attention for consistency is needed on this point. | Use of abbreviations is revised throughout the manuscript |
| Page 4, line 64 – should read ‘5-year mortality rate’ or ‘mortality at 5 years is…’ | Corrected |
| Page 4, line 84 replace wasn’t with was not | Corrected |
| Page 5, line 101- studies should read Studies… | Corrected |
| There is some inconsistency in the tense used in writing – should be written ideally in the past tense when describing the methodology throughout. | Revised throughout the manuscript |
| Page 5 – line 108 – should read ‘studies published only as conference abstracts (plural). | Corrected |
| Some correction to grammar is needed in the sentence on page 7, line 140. Also on line 156 – ‘two reviewers agreed upon a unique name for each outcome’. Other aspects of grammar would benefit from further attention within the manuscript with appropriate use of , and ; within sentence. At times, there is mixed use. | Corrected |
| Numbers less than 10 are generally written in full in academic writing. It would be worthwhile for the authors to check the guidelines for this journal on this point. | Numbers are revised throughout the manuscript |
| Incomplete sentence on page 9, line 192 – ‘While 23 compared one form of physiotherapy to another…’ | The sentence is now corrected. Page 11 line 203 |
| It is mentioned that there are 3 categories of airway clearance techniques, but these have not been described in the introduction or earlier in the methodology. | This sentence was removed from the text and details od airway clearance treatments are included in figure 2 which is referenced in the text. |
| The authors did not appear to include respiratory muscle training as a physiotherapy treatment, but it is not in the list for exclusion – can the authors clarify this point? Or can the authors justify why they included it under the rehabilitation title in Figure 2. | Respiratory muscle training is included as a physiotherapy treatment in table 1 and figure 2. |
| When the authors are specifying lung function as being the most common measure, it would be helpful in the text to know if this is static and dynamic measurements or dynamic measurements predominantly. | Lung function is explained in detail in box 1 and appendix 3. The phrase “, including static and dynamic Spirometric measurements,” was added. Page 12 line 235 |
| There are a number of abbreviations in Appendix 3 – should these be defined in a legend? | All abbreviations in appendix 2 and appendix 3 are now defined |
| Incomplete sentence on page 11, line 239 – ‘Which predicts a continuous problem of research waste in the future [35]’ Page 13, line 303 – typo – durnig should read during | Both corrected |
| Once an abbreviation is introduced, it is ideal if it can be consistently used – such as ACBT (written out in full again on page 13, line 306, but the abbreviation for this has been introduced earlier. | Sentence changed to: “the ACBT was investigated in four trials” |
